# Congruous Torsional Down Beating Nystagmus in the Third Position of the Semont's Maneuver in Patients Treated for Canalithiasis of Posterior Semicircular Canal Benign Paroxysmal Positional Vertigo: Its Significance and Prognostic Value

**DOI:** 10.3389/fneur.2020.00949

**Published:** 2020-09-15

**Authors:** Beatrice Giannoni, Vincenzo Marcelli, Ilaria Verdolin, Curzio Checcucci, Federica Pollastri, Rudi Pecci

**Affiliations:** ^1^Department of Neuroscience, Psychology, Drug's Area and Child's Health, University of Florence, Florence, Italy; ^2^Unit of Audiology Careggi University Hospital, Florence, Italy; ^3^Neurotology Unit, Ospedale del Mare, Naples, Italy; ^4^Department of Physics and Astronomy, University of Florence, Italy Careggi University Hospital, Florence, Italy

**Keywords:** benign paroxysmal positional vertigo treatment, Semont's liberatory maneuver, posterior semicircular canal, down beating nystagmus, torsional down beating nystagmus, canalolithiasis, liberating nystagmus, benign paroxysmal positional vertigo therapy outcomes

## Abstract

Due to its mechanical pathogenesis, benign paroxysmal positional vertigo treatment is mainly physical: when posterior semicircular canal is involved, Semont's maneuver is reported as one of the most effective liberating procedures. In the case of a canalolithiasis, the efficacy of the maneuver is corroborated by the appearance of some nystagmus findings during its performance. Liberating nystagmus, that can occur in the second position of Semont's maneuver and whose direction is congruous with the excitation of the affected posterior semicircular canal has proven to be a favorable prognostic sign. On the other hand, in clinical experience, we've frequently verified the appearance of another nystagmus during the execution of the maneuver: upon reaching the third position, when replacing the patient seated, a torsional down beating nystagmus, with the torsional component “congruous” with the stimulation of the vertical semicircular canals of the affected side, can often be appreciated. Such a sign can occur with or without having had the previous liberating nystagmus in the second position and is almost always associated with an intense vertigo and/or body pulsion. In this study, we describe the incidence and characteristics of the congruous torsional down beating nystagmus that can arise by assuming the third position of Semont's maneuver in a cohort of patients treated for posterior semicircular canal benign paroxysmal positional vertigo due to canalolithiasis. In the best of our knowledge, such a sign has never been described and explained before. On the basis of the pathophysiology and of the possible canal receptors stimulation during the different phases of Semont's maneuver, we formulated different hypothesis on how such a nystagmus can be generated. We observed that such a sign, when elicited, has a very good prognostic meaning for healing purposes, even better than that of liberating nystagmus. Therefore, congruous torsional down beating nystagmus should always be checked when performing Semont's maneuver because it could help in predicting success of physical treatment and in managing patients.

## Introduction

Posterior semicircular canal (PSC) benign paroxysmal positional vertigo (BPPV) is the most frequently diagnosed peripheral vestibular pathology (1–4). Being its pathogenetic mechanisms mostly those of canalo- (5) or cupulolithiasis (6), the therapy of choice is a physical one (7). When PSC is involved, the physical techniques for which it is reported the greater success are those of repositioning and those that take advantage of the brisk deceleration imposed to the otoconial mass. The percentage of short-term resolution obtained with the two types of maneuvers is similar, and the choice of one or the other technique is formulated according to patient's characteristics and operator's preference. The prototype of maneuvers exploiting the sharp deceleration imposed to the otoconial cluster is the Semont's liberatory maneuver (SLM) (8): a series of three rapid movements is performed in order to free the PSC from the mass of heavy particles, carrying the latter into the utriculus. During the execution of the maneuver, some nystagmus signs, which are significant for the success of the therapy, can appear: they can represent the manifestation of a correct movement of the cluster toward the exit from the canal. Namely, after the first movement of SLM, performed carrying the patient onto the pathological side, a mixed “Loading Nystagmus” (LoNy) will be generated: its direction (referring to the fast phase from here on) will be upward (toward the forehead) with the upper pole of the eyes beating toward the lower ear, thus in a counterclockwise and clockwise direction, due to the involvement of the right and left PSC, respectively. After the second movement of SLM, performed carrying the patient from the pathological onto the healthy side, a “Liberating Nystagmus” (LNy) with the same direction as the LoNy can be generated; this finding has been proved to be a good prognostic sign (9–11). Based on literature, the undisputed efficacy of SLM can be affirmed, with a short-term success rate of this physical therapy reaching about 80%. Moreover, the appearance of a LNy in the second position is related to an excellent prognosis (72–87%) in terms of resolution of symptoms and signs. From the personal practical experience, we have been able to notice that during the third movement of SLM, when returning the patient seated, a different nystagmus is often generated. When evident, the latter nystagmus is torsional vertical down beating on the whole, accompanied by a strong vertigo and retropulsion. Our hypothesis was that patients who manifest this finding have overall a faster resolution than those who do not or those who only have LNy. The purpose of our work was to verify the presence of nystagmus when taking the third position of the SLM in a cohort of patients treated for PSC canal lithiasis, to describe it, to hypothesize the mechanism by which it is generated, to quantify its impact and, above all, to evaluate its prognostic value for the resolution of PSC BPPV. A secondary objective of our study was to investigate the existence of other factors that may change the outcome of SLM and to evaluate the weight of each of them, in particular that of the time elapsed between the onset of the symptoms and the execution of the maneuver.

**Figure 1:**
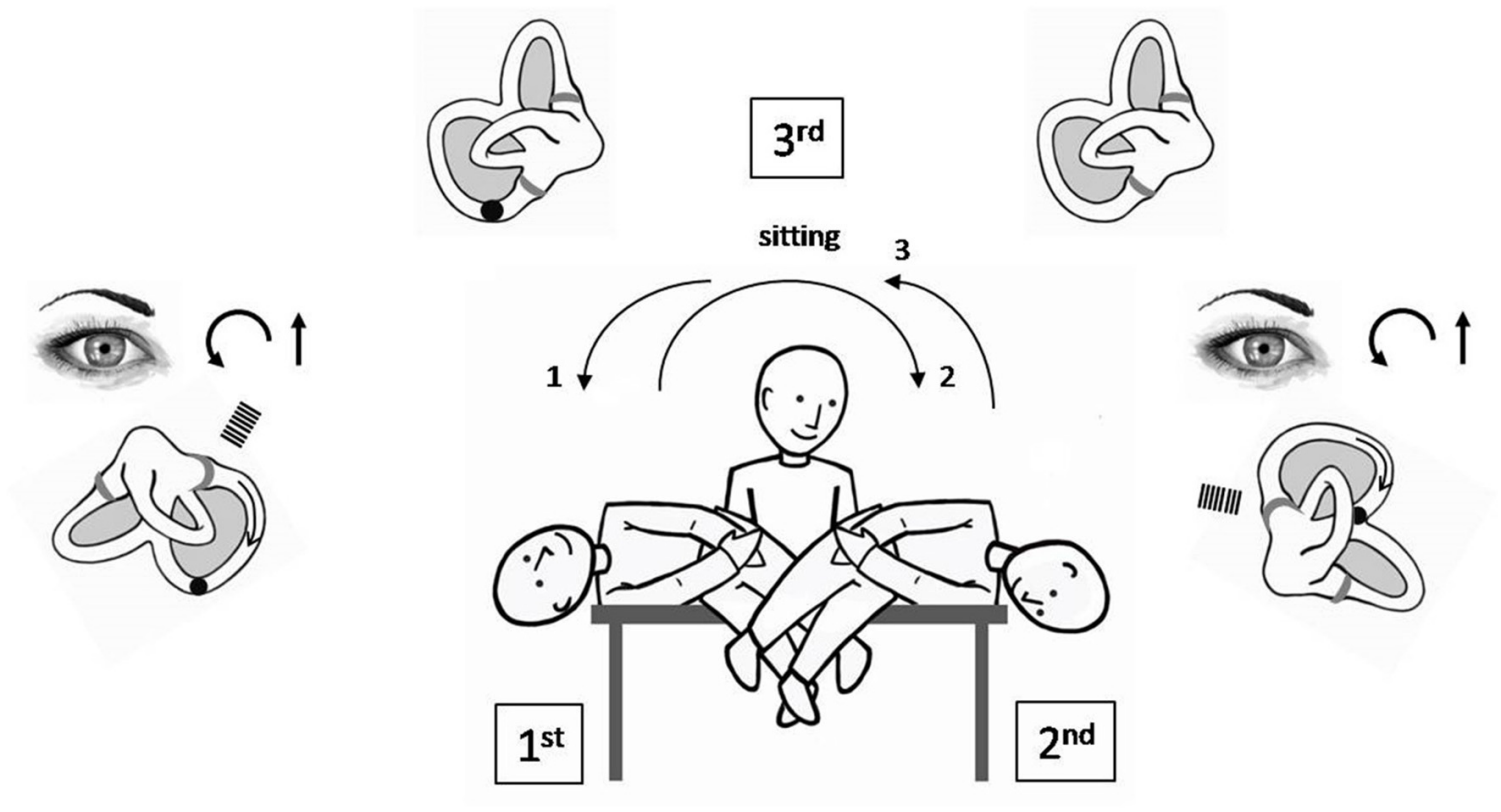
Semont's liberatory maneuver in the case of a right PSC BPPV canalolithiasis. Procedure starts having the patient seated in the center of the examination bed. The otoconial debris is deposited into the most declivous part of the PSC. (1) patient is rapidly brought from the sitting position toward the affected side maintaining the head turned 45° contralaterally and reaching a position of the head that is 20° under the horizontal (lower than the body) (1st position). The otoconial debris moves in the ampullofugal direction causing an ampullofugal endolymphatic flow, thus an excitatory discharge of the right ampullary nerve. Paroxysmal positional nystagmus occurs, being its fast phase counterclockwise and up beating (LoNy). (2) patient is quickly moved 220° toward the healthy side, keeping the head rotated as previously, and reaching a position of the head 20° lower than the horizontal, again (2nd position). Aim of the latter movement is to make the debris to continue its ampullofugal path toward the vestibule by briskly decelerating the head in reaching the second position. In this case, a paroxysmal positional nystagmus might occur, being its fast phase counterclockwise and up beating again (LNy). (3) patient is moved back to the sitting position (3rd position).

Since its first description, the procedure of SLM has been partially modified and simplified, with respect to the original, because of clinical and pathophysiological observations that have followed over years. The goal of SLM is to displace the otoconial debris from the ampullary portion of the PSC, where it is located when the patient is in the upright or sitting position, toward the utricle, passing through the non-ampullary tract of the canal, by taking advantage of the sharp deceleration that is imposed by means of specific brisk movements on the mass of heavy particles. SLM (Figure 1) starts having the patient seated in the center of the examination bed; the operator turns the subject's head 45° toward the healthy side in order to position the affected canal on the same plane on which the maneuver will be carried out. The first movement of the maneuver brings the patient from the sitting position to that of the pathological side (Figure 1, 1st). The latter shift should be performed with a high angular velocity and by reaching a position such that the body is displaced 110° with respect to the sitting position (the head being positioned 20° under the horizontal plane); actually, an only 90° displacement could be insufficient to move the particles in the declivous part of the PSC (12). In the case of a canalolithiasis, positioning the patient onto the pathological side causes the migration of the otoconial debris, by means of gravity, from the ampullary arm toward what it becomes the most declivous part of the PSC in the new position. Conversely, in the case of a cupulolithiasis, it is assumed that the cluster of particles weighs on the cupula, causing it to deflect toward the canal. In both cases, an excitatory stimulation of the PSC ampullary receptor takes place. The resulting nystagmus beats, therefore, upward and in a counterclockwise and clockwise direction (from the examiner's point of view from now on), due to the involvement of the right and left PSC, respectively. Once such an ocular movement, which we defined LoNy, is exhausted and waited about 45 s since the position is reached (12), the operator brings the patient onto the non-pathological side (Figure 1, 2nd) taking care to maintain the head still turned 45° toward it. The latter shift should have a 220° amplitude and should be executed with a high velocity. Indeed, for the second movement of SLM also, it has been demonstrated that reaching a position of the head that is lower than the horizontal is more effective in moving the cloth toward the utricle (12). With such a second movement, in the case of a canalolithiasis, the maneuver aims to move the otoconial debris still in the ampullofugal direction, toward the utricle. If such a displacement occurs, an endolymphatic flow is determined such as to give again an excitatory stimulation of the ampullary receptor; nystagmus thus generated beats upward and in a counterclockwise and clockwise direction, respectively, for the right and left PSC. The appearance of this finding, called LNy, is expressive of an effective ampullofugal movement of the heavy debris into the canal lumen, though it cannot provide any indication about the stretch covered by the otoconial mass into it, by means of the maneuver (13–15). The brisk deceleration obtained with the second movement of SLM could also lead to a backward path of the otoconial mass toward the ampulla, thus provoking an inhibitory discharge of the PSC ampullary nerve. The nystagmus so generated would have a direction opposite to that of the LoNy, being therefore down beating and with the torsional component directed clockwise and counterclockwise, respectively, for the right and left PSC. The appearance of such a nystagmus would have therefore a bad meaning with regard to the success of the maneuver (16). Even in the case of a cupulolithiasis, the sudden deceleration obtained with the second movement of SLM could theoretically give rise to two types of ocular movement; the first is a nystagmus with a direction similar to that of the LoNy, meaning that the heavy material has been detached from the cupula by flaunting it in the ampullofugal direction. The second is a nystagmus with a direction reversed with respect to that of LoNy meaning that the mass adhering to the cupula pulls it in the ampullopetal direction, thus determining an inhibitory discharge of the posterior ampullary nerve. In both a canalo or cupulolithiasis, a third possibility exists: when the patient is brought onto the second position of SLM no signs are highlighted. In this event, it is probable that there is no further movement of the particles inside of the canal because they could have already been brought out of it with the first movement or could have remained in the previous position (11, 17).

Once nystagmus in the second position is exhausted (or after about 45 s, if no findings is observed), the operator moves back the patient to the sitting position (Figure 1, 3rd).

Theoretically, during the latter movement, it can happen that:

- no further stimulation of any canal is produced because otoconial debris has already reached the utriculus; in such a case no endolymphatic flow and no nystagmus would be generated.
- debris moves backward into the PSC giving rise to an inhibitory stimulation of the corresponding ampullary nerve; in that case a torsional vertical down beating nystagmus would be generated with the torsional component opposite to that of the LoNy nystagmus;
- the otoconial cluster moves toward the vestibule, having already traveled the non-ampullary arm of the PSC; such a movement should therefore generate an endolymphatic flow into the common crus, toward the utricule. Nystagmus expected will be torsional vertical having the fast phase of both the linear and torsional components that represents the algebraic sum of the two vertical canals stimulation. Theoretically, stressing both vertical semicircular canals, should give rise to a mainly torsional nystagmus because the linear components would cancel each other while the torsional movements would sum. However, during SLM, it would be the position that each vertical canal reaches, together with the efficacy of the endolymphatic currents generated inside of them, that will origin the final ocular movement.

In literature, no author has dealt with describing the possibility of presenting and the characteristics of nystagmus findings in the third position of SLM, after having or not a LNy in the second one. Moreover, no study on the prognostic value of these signs have been published. Albera et al. only focused on the prognostic value of vertigo occurring in the last position of SLM (10). On the other hand, practice suggested us that the onset of a nystagmus in the third position of the SLM is a common occurrence and that, when present, it has a direction compatible with the simultaneous stimulation of two vertical semicircular canals of the affected side. In particular, nystagmus observed during SLM on return to central position is, in our experience, a torsional down beating nystagmus whose torsional fast phase is “congruous” with that of the LoNy (cTDBNy) and whose pathophysiological explanation will be given later. Furthermore, in everyday clinical practice, we had the feeling that patients presenting this finding, although suffering more from physical therapy, had a better and faster course than those who showed only the LNy.

## Materials and Methods

### Subjects and Clinical Methods

Our work deals with a logistic regression study, conducted at the Audiology Unit of Careggi University Hospital in Florence on a series of 55 selected outpatients suffering from PSC BPPV. Cases were collected during a 1-year period, between November 2018 and December 2019.

Subjects came to visit at various distances from the onset of symptoms, which made it possible to evaluate acute patients but also those who had long-lasting dizziness.

An accurate specialist and general history was collected for all patients, aimed at identifying the peculiar characteristics of BPPV, its origin, the presence of any other neuro-otological and/or systemic pathology capable of influencing the clinical picture.

All subjects underwent an otomicroscopic examination and audiometric and impedance testings. All patients were submitted to a bedside neuro-otological examination, including studying of the visuo-oculomotor systems (saccadic and smooth pursuit) and searching for gaze-evoked, rebound, spontaneous, positional, and positioning nystagmus. Nystagmus findings were observed with and without visual fixation, under Frenzel glasses or infrared video-oculoscopy. Vestibulo-oculomotor reflex function testings were also performed for all patients at different stimulation frequencies: head impulse test, head shaking test, and binaural bithermal calorics.

Since the presence of central vestibular signs had been a reason for exclusion from the sample, subjects did not undergo neuroradiological examinations, neither at the first evaluation nor at the control visit. Instead, these investigations were performed when the patients had not resolved BPPV in the foreseeable time or showed semeiological atypia during follow-up.

Patients who had the following inclusion criteria were selected: (a) diagnosis of a primitive idiopathic PSC BPPV; (b) geotropic positional torsional up beating nystagmus evoked by the Dix-Hallpike's positionings and showing the characteristics of a canalolithiasis of the PSC (i.e., nystagmus arising with latency, with a “crescendo-decrescendo” paroxysmal trend, with a duration <60 s and a direction reversal, with a lower amplitude, when returning to the sitting position); and (c) BPPV strictly involving a single canal.

The following exclusion criteria have been envisaged: (a) positional vertigo secondary to labyrinthopathy, surgery, or trauma; (b) concomitant diseases affecting the vestibular system or the inner ear; (c) positional nystagmus suggesting PSC cupulolithiasis (i.e., without latency, without crescendo-decrescendo trend, with a long duration, over 1 min, and without a clear reversal in the sitting position); (d) orthopedic, cardiological, neurological, or other systemic contraindications to the execution of the maneuver; and (e) presence of situations that could compromise the proper execution of the release maneuver, such as obesity, physical malformations, and orthopedic disorders.

Once a diagnosis of PSC BPPV was made according to the established criteria, patients were informed about the nature of their vertigo and the possibility of performing physical therapy. A procedure of SLM was then described together with the possible dizzying events occurring during the execution, as well as the postural and general sequelae that the maneuver could determine in the following days, even in the case of a positive result. Subjects were also advised of the intention to immediately verify the outcome of the SLM by repeating the diagnostic Dix-Hallpike's positioning.

During the performance of SLM, without using devices inhibiting fixation, we checked the presence of: (a) LoNy in the first SLM position; (b) LNy in the second SLM position; and (c) any nystagmus in the third SLM position and, in that case, we detected its qualitative, direction, and plane characteristics; in particular, attention was paid to the appearance of a vertical torsional nystagmus, with a direction compatible with the excitatory stimulation of one or both vertical canals, or with PSC inhibition. After a short period of rest (2–3 min), a suitable Dix-Hallpike's diagnostic retest was performed. During the latter positioning, the absence or presence of signs of PSC canalolithiasis, as well as their typology, was assessed.

All patients treated were scheduled to undergo a neurootological examination within a short time (max 10 days) and suggested to avoid abrupt movements on the vertical plane in the following 48 h, inviting them to sleep uplifted on the two nights following the maneuver.

Subjects were discharged without prescribing any further investigation if they were asymptomatic at the check-up, being recovery verified by the absence of findings; in case of persistence of PSC BPPV nystagmus, patients were again treated with the same maneuver or with a different physical therapy. For patients with vertigo refractory to therapy or with a persistence of vestibular signs other than those typical of PSC BPPV, an in-depth diagnostic procedure was planned.

Data were collected, updated, and archived in a database that served for clinical and statistical considerations.

The following further characteristics of the sample were considered: sex, age, affected side, time from the onset of symptoms, presence of a LNy, presence/absence and typology of nystagmus in a suitable Dix-Hallpike's positioning early retest, performed few minutes after SLM, and presence/absence and typology of nystagmus signs at the control visit.

### Statistical Analysis

The first exploratory model has been carried out to better understand the relationships between the nystagmus presence/absence in the second (liberating) and in the third position of SLM and the Dix-Hallpike's position retest. The Dix-Hallpike's retest has been considered as a dependent variable since it has been pointed out to be an early index of resolution of the clinical picture. Nystagmus evidence in Dix-Hallpike's retest has been codified with three possible outcomes: “*absent*,” “*excitatory*,” or “*inhibitory*,” where “*inhibitory*” describes those situations in which nystagmus has been generated by an ampullopetal stimulus. For this purpose, we inferred the parameters of a multivariate multinomial-logit model.

Then, the relationships with healing has been explored. In particular, we took a look both at the most predictive signs of the final resolution and at the possible intervenient effects among them. For the latter purpose, two nested multivariate logit model has been used.

In all the three models, we included four control variables to avoid overestimation of the relationships due to unconsidered intervenient effects. These four variables are: sex, age, affected side, and time (in days) from the beginning of the symptoms.

Estimated coefficients together with the related standard deviations and *p*-values are reported in Tables 1–**3** for models 1, 2, and 3, respectively.

**Table 1:** Model 1.

	**Response variable**	
	**Dix-Hallpike Nystagmus (ref. cat.: “excitatory”)**	
	**(“Absent”)**	**(“Inhibitory”)**
Age	−0.017 (0.028)	−0.033 (0.040)
Sex (“*man”*)	0.504 (0.849)	0.039 (1.207)
Affected side (“*left”*)	0.684 (0.931)	1.217 (1.198)
Symptoms onset	0.004 (0.006)	−0.007 (0.013)
LNy (“*present”*)	1.764 (1.086)	11.438^***^ (1.481)
cTVDBNy (“*present”*)	−0.225 (0.823)	−1.837 (1.183)
Observations	55	
Akaike Inf. Crit.	110.391	

Estimates of multinomial logit model for nystagmus findings on Dix-Hallpike diagnostic retest. ^*^p < 0.1; ^**^p < 0.05;

*** *p < 0.01*.

## Results

### Clinical Findings

From the analysis of the population examined, a different composition of the sample with regard to gender emerges: 35 out of 55 subjects were females (64%) and 20 males (36%). The age affected by PSC BPPV varied, in our cohort, from 39 to 93 years, average age 68.3 years.

Thirty-eight patients out of 55 (69.1%) presented a right PSC BPPV; the remaining 17 showed, instead, typical semeiological findings of a left one.

Concerning the time elapsed between the onset of symptoms and the diagnosis, the distribution of the patients examined is such that a large percentage was assessed at a relatively short distance, but the sample is composed for a conspicuous part also by subjects for whom the disease has been dated for a longer time. In particular, 24 patients were seen within the first week, 13 within 1 week to 30 days, and 18 subjects within a year from symptoms onset.

In the ipsilateral diagnostic Dix-Hallpike's position, all patients showed the typical nystagmus finding for an ampullofugal PSC stimulation with the peculiar characteristics of canalolithiasis.

Reaching the first position of the SLM, all the patients presented the LoNy, generated by the expected displacement of the otoconial cluster from the ampullary to the non-ampullary tract of the PSC; as explained, this is a vertical torsional nystagmus, with the characteristics of a finding justifiable with a canalolithiasis, the direction of which was upward (geotropic) and torsional counterclockwise and clockwise, for the right and left PSC, respectively.

Forty-nine/55 (89.1%) patients showed a nystagmus in the second position of SLM. In all subjects, this finding has been a LNy, which means that its direction was similar to that of LoNy, both for the vertical and the torsional component.

When reaching the second position of SLM, no findings occurred in the remaining 6/55 patients (10.9%). Absence of nystagmus is likely to be attributed to the lack of any further movement of the otoconial debris into the canal, after the declivous position is reached with the first movement. Therefore, not having any nystagmus in the second position has a neutral meaning until it is not associated with any subsequent finding.

However, none of the 55 subjects, in the second position of the SLM, showed a nystagmus indicative of an inhibitory stimulation of the PSC (apogeotropic), suggestive of a backward path of the clot in the ampullopetal direction.

On returning to a sitting position, with the third movement of the SLM, 27 out of 55 (49.1%) patients showed a vertical torsional nystagmus having the linear component directed downward (down beating, geotropic) and the torsional component “congruous” with the stimulation of the vertical canals of the affected side.

None of the latter 27 subjects witnessed the appearance of a nystagmus compatible with an inhibitory stimulation of the lonely PSC.

The appearance of signs during both diagnostic and therapeutic movements has invariably been associated with a corresponding patient's dizzying sensation.

Namely, very often the triggering of nystagmus with the assumption of the third position of the SLM was accompanied by a violent vertigo and retropulsion. In our study, however, we did not deal with examining the symptoms, but only the signs.

Among the 49 subjects who presented LNy in the second position of SLM, 34 (69.4%) had a negative Dix-Hallpike's early retest; eight out of these 49 patients (16.3%) had a torsional down beating, without latency and not paroxysmal nystagmus, compatible with a residual otoconial clot of the non-ampullary arm, which partially returned toward the ampulla by assuming such a position, thus causing an inhibitory stimulation of the PSC. The latter finding is not to be considered expressive of a negative result of the maneuver, but only of the persistence of some debris into the non-ampullary tract of PSC. These patients were dismissed without performing further early diagnostic retests or therapeutic maneuvers. Seven/49 patients (14.3%), despite having exhibited a LNy, still showed a nystagmus indicative of an excitatory stimulation of the PSC. Such a nystagmus was, therefore, suggestive of the permanence of a residual clot into the ampullary tract of the PSC.

Therefore, among the 49 patients manifesting a LNy, 69.4% (*n* = 34) had actually a negative Dix-Hallpike's retest, confirming the exit of the clot from the PSC non-ampullary arm; if we add to this population subjects who showed nystagmus of the inhibitory type at early retest (*n* = 8), the percentage of favorable verifications rose to 85.7%.

Six patients out of the total (10.9%) didn't show LNy: three subjects didn't manifest any nystagmus at the early retest, while the remaining three had a nystagmus, indicating the persistence of periampullary lithiasis. It is not superfluous to underline that, at the Dix-Hallpike's retest, none of these six subjects presented an inhibitory nystagmus.

**Figure 2:**
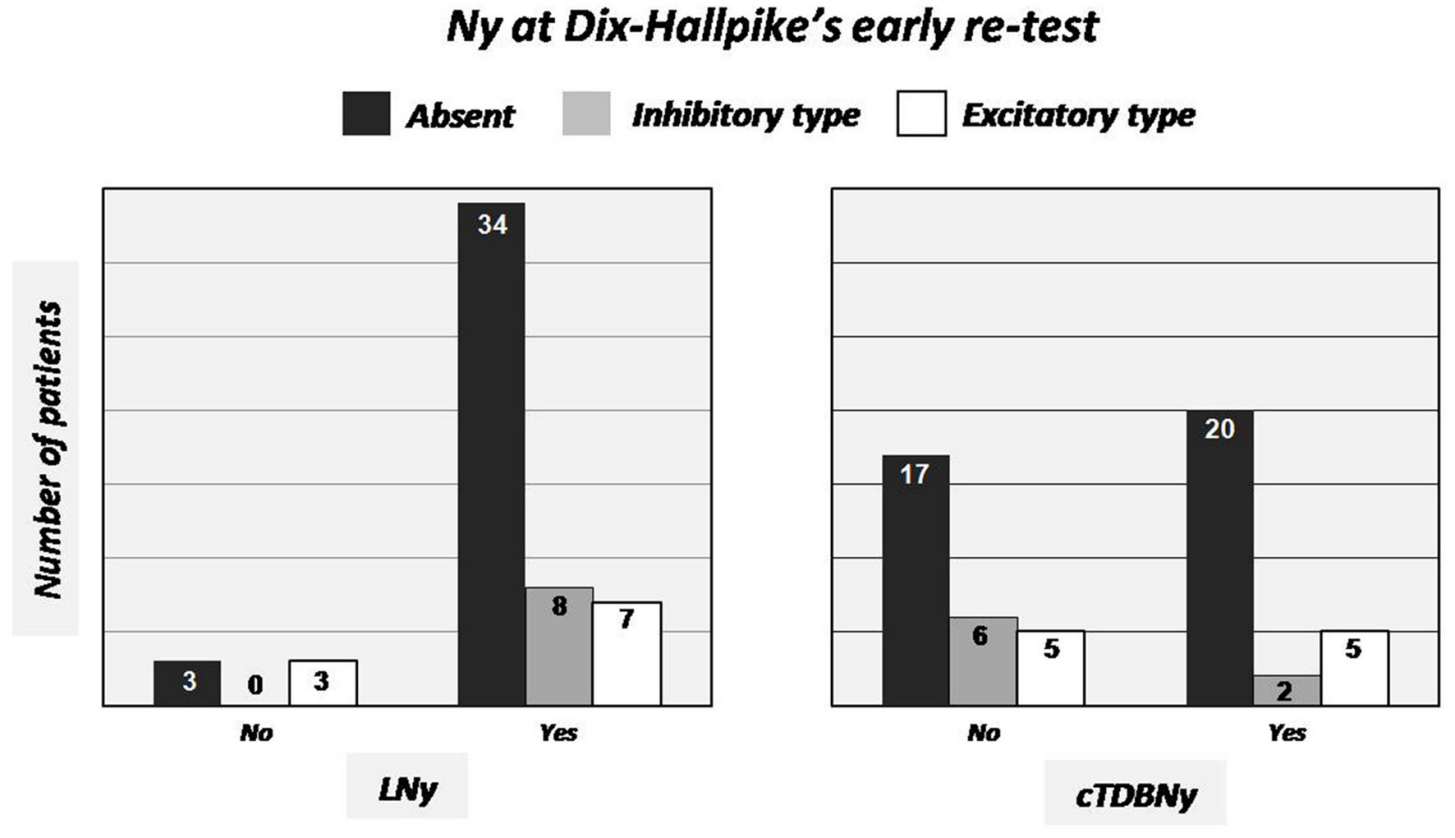
Graphic representation of the relationships existing between the absence/presence and typology of nystagmus at Dix-Hallpike's early retest in the case of LNy (left side) and cTDBNy (right side) evidence or not.

Figure 2 (left side) shows the above results with regard to the correlation between the presence/absence of LNy in the second SLM position and findings highlighted at the Dix-Hallpike's retest.

Within the series, we subsequently examined the relationship existing between the absence in the Dix-Hallpike's early retest and the previous appearance of a nystagmus on returning to sitting position with the third movement of SLM. As reported, the latter nystagmus, when elicited (27 out of 55 patients, 49.1%) was always of the torsional down beating type, with the torsional component “congruous” with stimulation of the vertical canals of the affected side (cTDBNy).

In 20 out of 27 patients (74.1%) presenting cTDBNy in the third position of SLM, the further Dix-Hallpike's retest was negative. This percentage increases to 81.5% if considering also patients (2/27) manifesting a weak, without latency and not-paroxysmal inhibitory nystagmus, likely due to an endolymphatic flow generated by the movement of a residual clot into the non-ampullary tract of the PSC, in the ampullopetal direction (inhibitory nystagmus).

Five out of the 27 (18.5%) showing cTDBNy, at early Dix-Hallpike's retest, still had a nystagmus indicative of a periampullary lithiasis (excitatory nystagmus).

Twenty-eight subjects out of 55 (50.9%) did not present any nystagmus moving back to the sitting position: 17 (60.7%) of them were negative at the Dix-Hallpike's verification, 6 (21.4%) had an inhibitory and 5 (17.9%) an excitatory type of nystagmus. Correlations between absence/presence of cTDBNy in the third position of SLM and Dix-Hallpike's early retest findings are represented in the graph of Figure 2 (right side).

**Figure 3:**
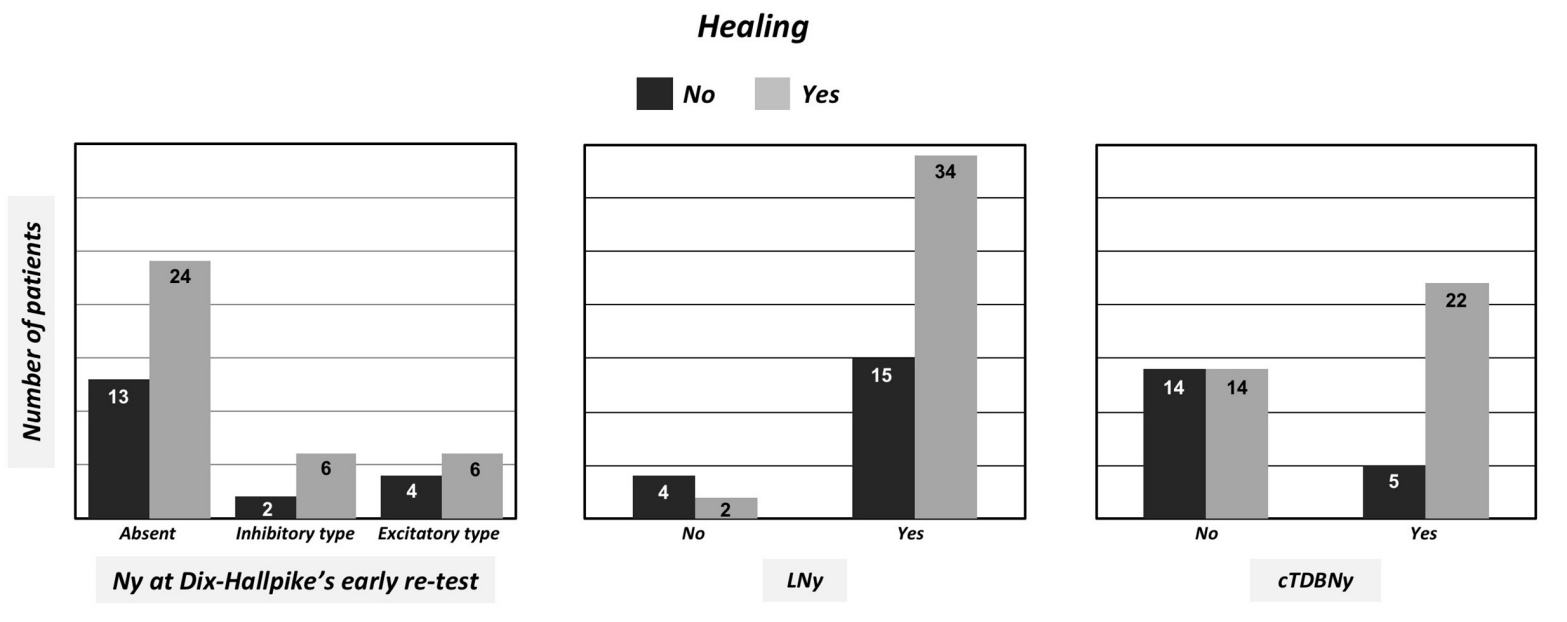
Graphic representation of PSC BPPV healing related to the absence/presence and typology of nystagmus at Dix-Hallpike's early retest (left side), to absence/presence of LNy in the second position of SLM (center), and to absence/presence of cTDBNy in the third position of SLM (right side).

The relationships existing between healing of the pathology, verified at the control visit, and the absence of signs at Dix-Hallpike's retest, was further investigated; in other words, we analyzed the concordance of immediate outcome of SLM with that verified at a distance. Among patients not having signs at an immediate control (*n* = 37), 64.9% (*n* = 24) were actually negative at distance. Adding to this quota also that of subjects who had nystagmus indicating PSC inhibition at the immediate verification test (6/8), it can be observed that the percentage of agreement between instantaneous and at distance positive results rose to 66.6%. However, 60% (*n* = 6) of the 10 patients having an immediate Dix-Hallpike's retest suggestive of persistence of a periampullary lithiasis, were also negative at the control visit (Figure 3, left side); five of these 10 patients had showed cTDBNy and, among them, three underwent resolution at the control visit.

We also took into consideration the relationships between the presence/absence of LNy and healing, regardless of the presence/absence of cTDBNy: 69.4% of patients with LNy went to recovery (34/49). Conversely, among those who did not present this finding (*n* = 6), only 33.3% (*n* = 2) were cured. These results are shown in the graph in Figure 3 (at the center).

Similarly, and this is the fundamental node of our study, we searched the correlations between the presence/absence of cTDBNy and healing (regardless the presence/absence of LNy). Among the 27 patients who experienced cTDBNy, even the 81.5% (22/27) had solved at the follow-up visit. On the contrary, among those who did not present cTDBNy (*n* = 28), only 50% (*n* = 14) went to healing (Figure 3, right side).

### Statistical Findings

Looking at the estimates of statistical models, whose single parameter interpretation, like the others models, has to be intended keeping fixed all the others, no meaningful changes in the odds-ratio of the immediate resolution, verified in the Dix-Hallpike retest, are produced by the presence/absence of cTDBNy (Table 1). On the other hand, the probability ratios between a Dix-Hallpike's retest, indicating inhibitory stimulus and that of the Dix-Hallpike's position positivity, significantly change (at a confidence level of 99%) when LNy sign is reverse. No other unitary variations in others variables seem to change the odds-ratios of the possible outcomes of Dix-Hallpike's position. In this scenario, we could hardly predict the considered immediate resolution of the disease.

However, when looking at the relationships of early retest findings with the definitive ones, we found good predictive capabilities for both LNy (0.05 < *p* < 0.1) and cTDBNy (0.01 < *p* < 0.05) (see Table 2). The probability ratio between positive and negative resolution is e^β5^ = e^1.732^ = 5.65 times higher for people who showed LNy than that of people with equal characteristics that did not show it. This proportion decrease to e^β6^ = e^1.496^ = 4.46 for people showing or not cTDBNy. The difference in the significance level has to be attributed to the higher variance of β_5_ parameter, possibly due to the unbalance in the number of patients with different sign with respect to LNy.

**Table 2:** Model 2.

	**Response variable**
	**Definitive resolution (*ref. cat.: “yes”)***
Age	−0.004 (0.025)
Sex (“*man”*)	−0.322 (0.666)
Affected side (“*left”*)	0.197 (0.728)
Symptoms onset	−0.004 (0.004)
LNy (“*present”*)	1.732^*^ (1.022)
cTVDBNy (“*present”*)	1.496^**^ (0.674)
Observations	55
Akaike Inf. Crit.	74.650

*Estimates of logit model for the definitive resolution test. In this specification, findings in Dix-Hallpike diagnostic retest are not included in the explanatory variables*.

* *p < 0.1*;

** *p < 0.05*;

^***^p < 0.01.

**Figure 4:**
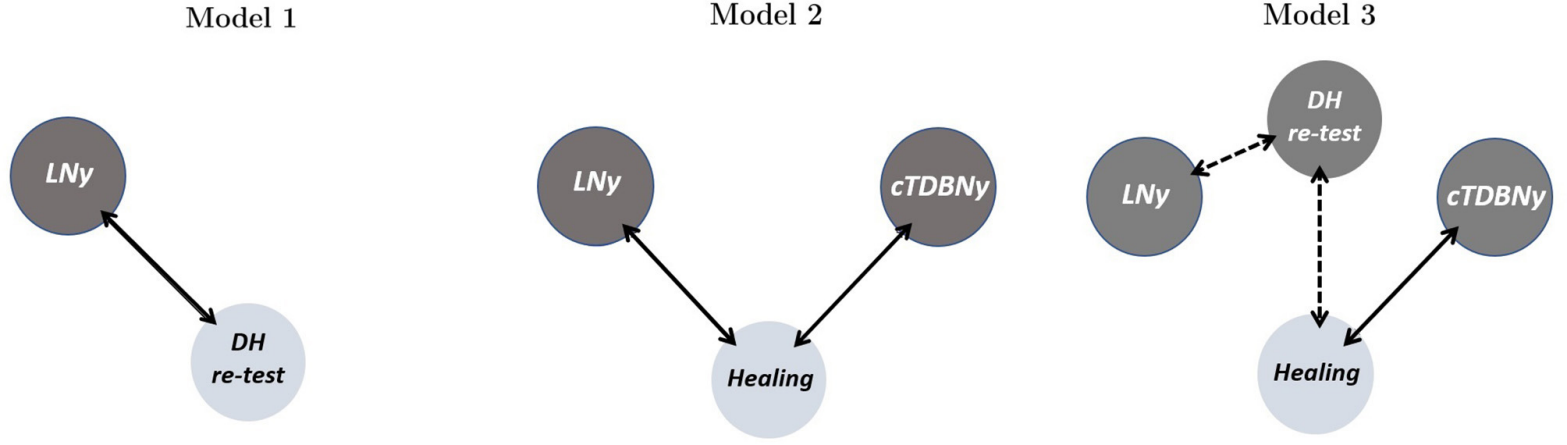
Relationships existing among nystagmus findings at early Dix-Hallpike's retest, at short-term control visit and during SLM in different positions. Colors separate the sides of model specifications: light gray for dependent variables and dark gray for explanatory variables. Solid line arrows represent statistically significant relationships, as found by the related model, wherever dashed line arrows signal those deduced from estimates changes between the three models (see Tables 1–3), literature and clinical experience.

The third model (whose estimates are resumed in Table 3) underlines the predictive role of cTDBNy in healing. The inclusion of Dix-Hallpike's retest nystagmus variable in the model does not affect its significance; instead, the estimated variation of the odds increases from 4.46 to 5 times higher for people who showed a cTDBNy with respect to people not manifesting it, but with equal values of the other covariates. Despite the non-significance of Dix-Hallpike's retest findings in changing the probabilities of healing may be due to an excessive fragmentation of data, it is worth to notice the loss in significance, for the (log) odds-ratio, of LNy. This result is interesting because it highlights an intervenient action of the Dix-Hallpike's retest variable, which therefore can affect both the definitive healing and the LNy manifestation. This thesis is also validated by model 1 results (Table 1) that show the relationship between LNy and Dix-Hallpike's retest nystagmus. Figure 4 represents the relationships highlighted by the estimates of the three models. Solid lines are for statistically significant relationships, wherever dashed lines are deduced from estimates changes between the three models, literature, and clinical experience.

**Table 3:** Model 3.

	**Response variable**
	**Definitive resolution (*ref. cat.: “yes”)***
Age	−0.004 (0.025)
Sex (“*man”*)	−0.316 (0.672)
Affected side (“*left”*)	0.228 (0.759)
Symptoms onset	−0.004 (0.004)
LNy (“*present”*)	1.620 (1.103)
cTVDBNy (“*present”*)	1.611^**^ (0.697)
DHNy (“*absent”*)	−0.046 (0.863)
DHNy (“*inhibitory”*)	0.661 (1.180)
Observations	55
Akaike Inf. Crit.	78.091

*Estimates of logit model for the definitive resolution test. In this specification, findings in Dix-Hallpike diagnostic retest are added to the explanatory variables*.

^*^p < 0.1;

** *p < 0.05*;

^***^p < 0.01.

Finally, regarding the four control variables included in all models, we notice how they don't seem to affect neither the Dix-Hallpike's early retest findings nor healing, meaning that the SLM outcome could not be influenced by age, sex, affected side, or interestingly, by the time elapsed between the symptoms onset and the execution of the maneuver.

The analysis of the first statistical study model shows that there is no significant relationship between cTDBNy in the third SLM position and the immediate resolution, verified by the absence of nystagmus in the Dix-Hallpike's retest (model 1). However, the prognostic role of cTDBNy in determining healing is evident. In fact, the latter sign appears to have good predictive capability for healing, with a *p* < 0.05 (model 2). Furthermore, this datum maintains significance also introducing nystagmus in Dix-Hallpike's retest among the explanatory variables under examination (Table 3, model 3).

LNy appearance is also significant for the resolution of the pathology, even if with a lower weight (*p* < 0.1) compared to the cTDBNy in the third position of SLM (model 2). Furthermore, by including among the explanatory variables the absence of nystagmus in Dix-Hallpike's retest, it can be observed how LNy loses significance, due to the interaction effect between the variables (model 3). Both of these aspects can be explained by the sample analysis of the statistical study: in fact, there is no homogeneity between the appearance (49 patients) and the absence (six subjects) of LNy in the second position. Consequently, the weight of the appearance of LNy is less influential than that of cTDBNy in the third position, in which there is a more homogeneous subdivision between the groups (27 patients have nystagmus, 28 patients do not).

Regarding the weight of Dix-Hallpike's retest findings in predicting healing, the absence of nystagmus was not particularly useful, at least regarding the examined patients (model 3).

In the last instance, going into the weight of the explanatory variables under examination, they are not related to the resolution of the clinical picture; especially as regarding the days between the onset of symptoms and the execution of the maneuver, statistical significance cannot be established.

## Discussion

The analysis of demographic data shows a substantial alignment with what reported in the literature regarding typical PSC BPPV. This feedback confirms the appropriateness of the inclusion and exclusion criteria adopted in selecting our case studies.

Moreover, in our cohort, PSC BPPV affects the right side more frequently. Therefore, in examining our patients we took care to test, in order, the left Dix-Hallpike's before the analogous positioning on the right; this behavior allowed us to carry out a complete examination always limiting patient's discomfort. Furthermore, thanks to selection criteria, we have never encountered any problem in performing all the required movements, nor have we ever been forced to suspend the examination due to patient's neurovegetative or other systemic symptoms.

Concerning the period of time elapsed between the onset of the disease and our diagnosis, it can be observed that many of the subjects in our series have been assessed in the acute or subacute phase (within 7 days). This certainly happened because our operating unit works in close collaboration with the emergency department, which sends us dizzying patients within a short time by means of a preferential path. It also happened that patients with recently onset vertigo came to our observation for a first evaluation, sent by the attending physician. On the other hand, there are not even a few patients who experienced long-lasting dizziness; among these there are subjects who had suffered in the past from BPPV and returned to visit because of a remote relapse or even individuals who carried out regular hearing checks.

Considering our sample, with regard to the relationship between the time of treatment and symptom's onset, we cannot definitely exclude that the resolution of the picture occurred spontaneously; in fact, the percentage of resolution of the pathology in cases seen at a shorter distance after the onset of symptoms is higher (27/37, 73%) than that found in patients for which diagnosis was made later (9/18, 50%). In any event, the probability of a spontaneous recovery rate as reported in literature is 20% for patients evaluated within 30 days, which is therefore much lower than the one we found for our patients.

From the analysis of our data, it is very clear that the lesser or the greater precocity of treatment does not significantly affect the final outcome; in fact, 27 out of 37 patients with recent vertigo (within 30 days) resolved at the first check but also nine out of 18 subjects with long-standing vertigo (30 days to 1 year) had an excellent control of the disorders with just one treatment. This result seems significant to us because it suggests that this type of vertigo does not tend to worsen over time and probably does not significantly alter labyrinthine metabolism nor the physiological mechanisms, allowing reabsorption of otoconial debris within the vestibule. From a practical point of view, this feedback indicates that, when faced with a patient who has symptoms and signs of PSC BPPV, it is possible to defer treatment for a few days because it will not compromise healing of the pathology. Treatment, if necessary, can be procrastinated to a more favorable time when subject's general conditions are better and patient, if it is useful, can come to visit pharmacologically prepared in order to control neurovegetative symptoms and even the anxiety that this disorder and its therapy can, sometimes, cause.

To confirm this, from our data it also emerges that, even in those few cases in which physical therapy has not been successful at the first session, an association with the late diagnosis/intervention can be seen.

All subjects examined and treated for BPPV had a semeiological picture suggestive of a PSC “canalolithiasis.” This is a significant premise because in the presence of otoconial debris free to move within the canal lumen, by observing the eye movements, we can predict the path covered by the otoconial clot as a result of changes in head and body position performed for diagnostic or therapeutic purposes (18). On the contrary in cupulolithiasis, while the behavior of the “heavy” cupula during diagnostic movements is quite clear, it is not equally understood what happens to this receptor following therapeutic shifts. To evaluate the findings highlighting during SLM, it was therefore essential to select subjects whose nystagmus pattern indicated a canalolithiasis rather than a cupulolithiasis pathogenetic mechanism.

Although necessary in a pilot study, the exclusion of cases in which nystagmus suggested cupulolithiasis as a pathogenetic mechanism could be actually a bias in interpreting mechanisms underlying cTDBNy generation in the last step of SLM. Further studies should be needed including even cupulolithiasis cases in order to check if cTDBNy is found as frequently as in canalolithiasis. If this is does not happen, as it is most likely in our opinion, the observation will further confirm our pathophysiological hypotheses.

During the therapeutic phase, we observed nystagmus in fixation condition; visual fixation, in fact, certainly does not succeed to cancel or to significantly inhibit the large nystagmic movements that are generated during liberating maneuvers. This is all the more so in the case of nystagmus generated by vertical semicircular canals, because their torsional component is not affected by fixation, since the ocular movement takes place around the anteroposterior axis of the ocular globe and therefore does not result in the slipping of the image on the fovea and in the consequent retinal error responsible for fixation system activation.

Canalolithiasis mechanism has been confirmed also by findings observed during SLM; actually, in the first position of SLM, all patients manifested the LoNy, which was similar to the one evidenced at the time of diagnosis. The linear and torsional components of LoNy were perfectly consistent with the excitatory stimulation of the of the pathological side on which the first movement of the SLM was performed.

The success of SLM has been ascertained by checking the disappearance or a positive modification of nystagmus in the Dix-Hallpike's early retest. It could be rightly observed that the time period that elapses between the execution of the release maneuver and the retest is short and that therefore the negativity of the maneuver is attributable to the refractory period; in reality, we believe that in the case of such short albeit intense nystagmus such as those typical of the CSP VPPB, the duration of such refractory period cannot reasonably exceed 3 min. Confirmation of the therapeutic success and therefore healing of the clinical picture was further verified by a follow-up visit carried out a few days after physical treatment.

Running SLM has never converted PSC BPPV into one interesting other semicircular canals thus confirming the lower tendency of deceleration techniques, with respect to repositioning ones, to determine a “canal switch” (19). A high percentage (65.5%) of patients met with resolution after SLM; this finding is absolutely consistent with what has been reported by other authors (9, 20–25). Moreover, 89.1% of our patients presented a LNy in the second position of the SLM; also, this figure is absolutely in line with what has been reported previously. LNy is indicated as a marker of success of the SLM and is correlated with the healing of the pathology. Soto Varela et al. (11) reported that 81% of their patients showed a LNy during the maneuver and healed. In our cases too, almost 70% (69.4%) of the patients who showed this finding were healed after the first maneuver. Therefore, LNy must be considered significant for prognostic purposes. So, as far as the success of therapeutic maneuvers is concerned, we can say that we have performed SLM in correct and effective manner. However, it should be noted that one-third of those few subjects who did not show LNy still healed at the first check-up. We therefore agree with what reported by Soto Valera et al. (11), that the absence of LNy is not necessarily linked to the failure of the therapy. The absence of such a finding is probably related to the lack of a further displacement of the otoconial cluster into the canal lumen after the initial one, achieved during the first phase SLM; therefore, it has neither a negative nor a positive meaning, with regard to the success of therapy, until it is not linked with further eventual events. The highlighting of an apogeotropic nystagmus in the second position of SLM, would have been, instead, a negative prognostic sign, because indicative of a backward path of the otoconial cluster, toward the ampulla. Such an event has never been observed in our case studies.

Conversely, LNy was linked neither to the absence nor to the finding of a torsional down beating nystagmus (apogeotropic) in the Dix- Hallpike's early retest. In other words, LNy did not clearly relate with the apparent early resolution.

From a statistical point of view, LNy seems to be correlated with the type of response at the Dix-Hallpike's retest; actually, LNy alters the probability ratios between the occurrence of an excitatory and an inhibitory nystagmus and the same it almost surely does with the probability ratios between the occurrence of an inhibitory nystagmus and a negative Dix-Hallpike's retest. On the contrary, LNy does not seem to alter the probability ratios between the occurrence of an excitatory nystagmus and a negative Dix-Hallpike's retest.

In addition, 35% of the patients who showed a favorable Dix-Hallpike's retest were still symptomatic and presented findings at a later check. This means that neither early negativity binds to the actual resolution of the picture nor the LNy is linked to a favorable early retest.

Rather, from the statistical analysis, it emerges that Dix-Hallpike's retest findings are not correlated with healing, while LNy is correlated both with Dix-Hallpike's retest results and with healing.

To the question of why sometimes the appearance of the LNy does not ensure the negativity of the early Dix Hallpike's retest it could be asked that maybe the second movement of the SLM effectively moves the debris in the ampullofugal direction but not enough to travel the whole canal; the retest positioning therefore could move the residual debris again in the ampullofugal direction.

With the third movement of SLM, which brings the patient back to the sitting position, in about 50% of our cases, we have witnessed a nystagmus whose appearance has been reported previously only in a very few patients (8/113). However, nystagmus reported by others was not described in detail, not being the subject of deepening in that study (10).

**Figure 5:**
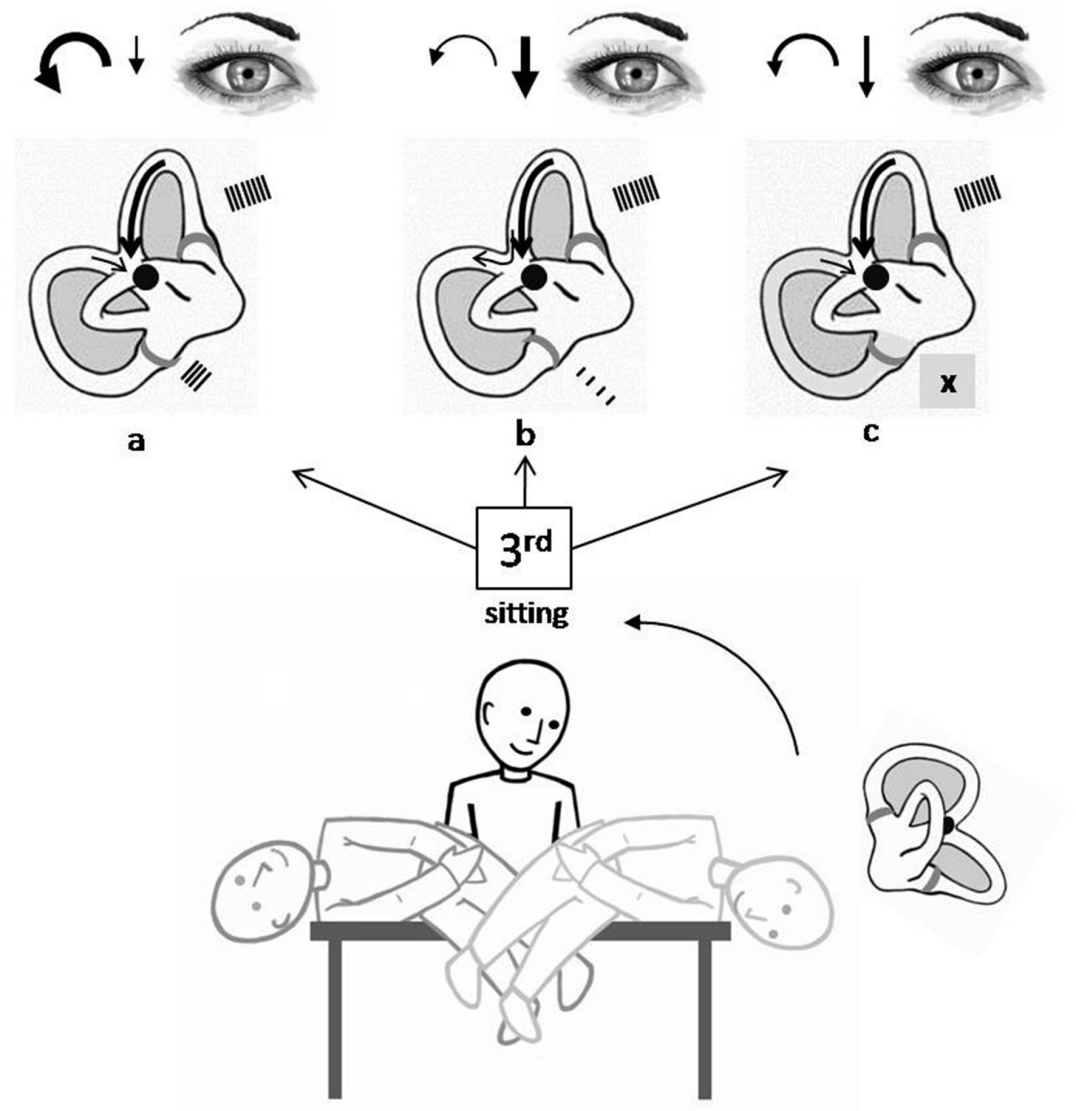
Possible mechanisms of cTDBNy generation in the third position of SLM performed for right PSC BPPV canalolithiasis. **(a)** The descent of the otoconial clot along the common crus provokes an ampullofugal endolymphatic flow, thus an excitatory stimulus, both into the ASC and PSC. Due to the final position reached by the posterior arm of ASC, more vertical than that of the non-ampullary arm of PSC, the outcoming ampullofugal endolymphatic flow is thought to be stronger in ASC than in PSC. Congruous TDBNy has a large counterclockwise and small down beating fast phase (algebraic sum of the consensual torsional and opposite vertical components deriving from the excitation of the two vertical canals). **(b)** The descent of the otoconial clot along the common crus provokes a strong ampullofugal endolymphatic flow into the ASC and a weak ampullopetal reflux into the PSC. Congruous TDBNy has a small counterclockwise and a large down beating fast phase (algebraic sum of the opposite torsional and consensual vertical components deriving from the excitation of ASC and inhibition of PSC). ASC contribution to cTDBNy is greater because of Ewald's second law and of its vertical orientation into the final position. **(c)** The descent of the otoconial clot along the common crus provokes an ampullofugal endolymphatic flow into both the vertical semicircular canals. However, PSC does not contribute to cTDBNy generation because its receptor is still in a refractory period, after being strongly excited in reaching the second position of SLM (and having generated LNy).

Nystagmus that we evidenced is a torsional vertical nystagmus, mandatorily indicating its origin from the vertical semicircular canals. This nystagmus, in our case study, had the linear component directed downward and the torsional fast phase effectively “congruous” with the stimulation the vertical canals of the pathological side. Referring to the torsional component, we've therefore called it “congruous torsional down beating nystagmus” (cTDBNy). This finding appeared almost without latency with respect to the assumption of the sitting position, it was often of remarkable amplitude, it had a relatively short duration, and almost always a clear paroxysmal trend. Once again, these characteristics are typical of the movement of free debris inside of the canal lumen. In fact, Squires et al. (26) have shown that heavy particles moving within the semicircular canal, without touching the walls and without passing through dilatations, produce this type of ocular movement. Symptoms accompanying cTDBNy were noteworthy and often associated with a clear retropulsion; it was our concern, in fact, to accompany, support, and contain the patient in returning to the sitting position precisely to avoid that the postural reaction could be harmful to the subject. With the third movement of SLM, a position is reached in which the posterior arm of the ASC is located in a maximum vertical position and therefore parallel to the gravitational vector. An otoconial cluster moving in the ampullofugal direction from the non-ampullary arm of the PSC would enter the common crus, thus provoking the aspiration of its endolymphatic column. Due to the extremely vertical position reached by the ASC posterior arm when sitting with respect to that taken by PSC non-ampullary tract, the aspiration of the endolymphatic column is likely to be much more effective in determining the excitation of the ASC with respect to that of PSC. ASC excitatory stimulus gives rise to a nystagmus beating with the torsional fast phase counterclockwise and clockwise, respectively, for the right and left CSA and, with the linear fast phase, downward. Although to a lesser extent, the same endolymphatic flow due to the passage of the cluster into the common crus may also be ampullofugal into the PSC, thus generating a nystagmus with the torsional component of the fast phase similar to that of ASC but with the linear fast phase directed upward. Due to the above anatomical considerations about the position of PSC non-ampullary arm, when seated, the contribution of the latter canal to the final ocular movement is likely to be weaker. Assuming these endolymphatic dynamics, the nystagmus observed on returning to the sitting position must be characterized by the algebraic sum of the torsional components (equal for the two vertical canals) and that of the two linear components (down beating for ASC and up beating for PSC). For what is previously explained, it will be generated a predominantly torsional nystagmus, congruous, with this component, to the excitation of the vertical canals of the affected side and down beating for the prevailing linear component due to ASC excitation (Figure 5, 3rd, a). Indeed, it could be even hypothesized that, due to the spatial orientation of PSC non-ampullary arm in the third position of SLM, the otoconial debris descending into the common crus may also create an ampullopetal reflux of endolymph from the ASC into the PSC, thus causing a weak inhibitory stimulus of the posterior ampullary receptor. The latter endolymphatic flow into the PSC would give rise to a nystagmus having the torsional component opposite to that caused by ASC excitation, but the linear one consensually directed. In this hypothesis, despite the reciprocal cancellation of the torsional components, the resulting nystagmus would be evenly torsional, because of the prevalence of the contribution of the excitation of the ASC (Ewald's second law). Moreover, nystagmus would be maximally vertical down beating because of the addition of the two linear components due to the opposite stimulations of the vertical canals (Figure 5, 3rd, b).

A third hypothesis can be formulated to explain the occurrence of a cTDBNy in the third position of SLM: it could happen that, in assuming such a position, the only stimulated canal could be the anterior one. That would happen because the PSC ampullary receptor would find in the refractory period after having discharged because of the ampullofugal endolymphatic current generated with the second movement of the maneuver (and originating LNy) (Figure 5, 3rd, c). Against this assumption is the occasional occurrence of cTDBNy also without having observed a LNy.

In all the three hypotheses, neither of which excludes the other, cTDBNy, that we have observed in half of our patients at the end of SLM, should be considered with a “liberating meaning” being the manifestation of the migration of the otoconial cluster correctly out of the canals.

We, therefore, wanted to verify the association of cTDBNy with the positive outcome of the maneuver, at distance and in the immediate. Indeed, 22 (81.5%) out of 27 patients who had cTDBNy were healed at the control visit while only 14 (50%) of the 28 who did not manifest it went to recovery. Such a clear-cut result, statistically supported (0.01 < *p* < 0.05), can only mean that the manifestation of a cTDBNy is related to an escape of the otoconial cluster from the canal and the common crus; this finding is effectively explained with the models described above, in which, according to the mechanism of canalolithiasis, the progressive movement of the cluster in this direction is envisaged. The abrupt retropulsion, so often observed as a reaction to the appearance of cTDBNy, could be the compensatory postural reaction to the down beating ocular movement, which occurs just when the body falls forward. Our results significantly differ from those reported by Albera et al. (10) in the only previous survey that deals with this problem; this discrepancy could be attributed to the small number of patients for which this finding has been described in that series.

On the contrary, the appearance of cTDBNy was not statistically related to the subsequent absence of findings with the early Dix-Hallpike's retest. However, if we evaluate numerical absolute values, we realize that among patients showing a cTDBNy, 81.5% have manifested, at the retest, findings indicative of total or partial release of the canal.

By concluding, in the clinical practice of a second/third-level neuro-otological center, diagnosis of PSC BPPV and its therapy are a consistent part of everyday activity. The evaluation and treatment of such a large number of patients, in various stages of acute illness, involves the observation of peculiar characteristics both of the specific clinical picture and of the events that may occur during physical therapies.

By performing SLM for PSC BPPV likely due canalolithiasis we have often found that, in addition to the LNy described in the second position, a further nystagmus can occur with the third movement of the maneuver, reaching the sitting position. The latter nystagmus is vertical and torsional, the linear fast phase being directed downward and the torsional component “congruous” with the stimulation of the two vertical canals, or at most just one (cTDBNy). As far as we know, this nystagmus has never been described and explained in the literature.

We wondered how this finding could be generated from a pathophysiological point of view, whether it corresponded to a more effective movement of the otoconial cluster toward the utriculus and therefore if this manifestation was synonymous of a better prognosis.

The working hypotheses were confirmed by the clinical data and the results of the statistical analysis. The cTDBNy is statistically correlated with a good prognosis as for short-term resolution of PSC BPPV, at least that supported by a canalolithiasis. Although indicative of good results, LNy appears to have a lower prognostic value than that of the cTDBNy.

The better therapeutic outcomes are neither linked to the early retest results, immediately verified after SLM, nor with the time of the onset of the disease.

From a practical point of view, therefore, in the case of a patient suffering from PSC BPPV, the correct therapeutic behavior can be performing SLM at the time of diagnosis and checking the appearance of the cTDBNy in the third position; if this finding occurs, it is very likely that patients will be free from the disease and that they probably should not undergo other treatments.

It is not necessary to immediately verify the outcome of SLM, thus avoiding the patient's fear of recurrence of symptoms and the possible mobilization of otoconial storage, which, in turn, may result in a return of the canaliths into the canal lumen and to the nullification of the result obtained. If SLM ends with cTDBNy and vertigo, patients can be reassured about the probable resolution of the disease and scheduled for a follow-up visit within some days. Patients can even decide themselves on returning to control basing on subjective symptoms. This also streamlines outpatient logistics by creating assessment possibilities for more urgent patients.

For what emerged from our results, it will therefore be possible to limit the post-maneuver restrictions favoring the resumption of natural movements as soon as possible, also in order to prevent the patients from continuing avoidance strategies, which instead would “paralyze” and predispose them to failure of readaptation.

It therefore seems significant to us to have identified a clinical sign, never systematically described previously, frequently highlighted during correctly performed SLM, which is simple to evaluate, even without particular technological aids, and which has a great prognostic value, even higher than that possessed from other signs equally frequently found.

## Data Availability Statement

The raw data supporting the conclusions of this article will be made available by the authors, without undue reservation.

## Ethics Statement

Ethical review and approval was not required for the study on human participants in accordance with the local legislation and institutional requirements. Written informed consent for participation was not required for this study in accordance with the national legislation and the institutional requirements.

## Author Contributions

BG conceived the study, provided for patient's recruitment, wrote the paper, and collaborated in hypothesis formulation. VM collaborated in study design, in hypothesis formulation, in writing, and commenting in the paper. IV collaborated in patients' recruiting and filling in the database. CC provided for statistical methods and evaluation of data. FP provided for filling in the database, collaborated in patients' recruiting, paper writing, and commenting. RP collaborated in conceiving and planning the study, in patients' recruiting, in writing the paper, and in hypothesis formulation. All authors contributed to the article and approved the submitted version.

## Conflict of Interest

The authors declare that the research was conducted in the absence of any commercial or financial relationships that could be construed as a potential conflict of interest.
